# Oxytocin restores cognitive function and attenuates neuroinflammation in chronic sleep-deprived aged rats

**DOI:** 10.3389/fnagi.2026.1742343

**Published:** 2026-02-10

**Authors:** Hany A. Elkattawy, Mohamed El-Sherbiny, Nehal Elsherbiny, Reham M. Wahid, Sherein F. El-Sayed, Hanan A. Henidi, Randa El-Gamal, Sultan Alshehri, Syed Mohammed Basheeruddin Asdaq

**Affiliations:** 1 Department of Basic Medical Sciences, College of Medicine, AlMaarefa University, Diriyah, Riyadh, Saudi Arabia; 2 Department of Pharmaceutical Chemistry, Faculty of Pharmacy, University of Tabuk, Tabuk, Saudi Arabia; 3 Department of Medical Physiology, Faculty of Medicine, Zagazig University, Zagazig, Egypt; 4 Department of Research, Natural and Health Sciences Research Center, Princess Nourah bint Abdulrahman University, Riyadh, Saudi Arabia; 5 Department of Medical Biochemistry and Molecular Biology, Faculty of Medicine, Mansoura University, Mansoura, Egypt; 6 Department of Medical Biochemistry, Horus University- Egypt, New Damietta City, Egypt; 7 Department of Pharmaceutics, College of Pharmacy, King Saud University, Riyadh, Saudi Arabia; 8 Department of Pharmacy Practice, College of Pharmacy, AlMaarefa University, Dariyah, Riyadh, Saudi Arabia; 9 Research Center, Deanship of Scientific Research and Post-Graduate Studies, AlMaarefa University, Dariyah, Riyadh, Saudi Arabia

**Keywords:** apoptosis, cognitive function, neuroinflammation, neuroprotection, oxytocin, sleep deprivation

## Abstract

**Background:**

Sleep is a fundamental biological process essential for maintaining mental, emotional, and physical health. Chronic sleep deprivation (SD), particularly in aging, is associated with oxidative stress, neuroinflammation, apoptosis, and cognitive and behavioral impairments. Oxytocin, a hypothalamic neuropeptide with reported antioxidant and anti-inflammatory properties, may modulate stress-related pathways; however, its role in mitigating SD-induced brain alterations remains unclear. This study investigated whether peripheral oxytocin administration influences brain and behavioral changes induced by chronic SD in aged rats.

**Methods:**

Male Sprague Dawley rats aged 20–24 months were divided into four groups: control, oxytocin-treated, SD, and SD with oxytocin treatment. Chronic SD was induced using a modified multiple platform method. Behavioral assessments were conducted to evaluate locomotor and exploratory activity. Serum cortisol was measured as a systemic stress marker, while brain tissues were analyzed for oxidative stress and inflammatory markers. Gene expression of Psen1 and Htr2a, oxytocin receptor protein levels, and histopathological changes, including gliosis and apoptosis, were also evaluated.

**Results:**

Chronic SD resulted in significant impairments in locomotor and exploratory behaviors, elevated serum cortisol levels, and increased oxidative stress and inflammatory markers. SD also altered the expression of Psen1 and Htr2a, reduced oxytocin receptor protein levels, and was associated with histological evidence of gliosis and apoptosis in brain tissues. Peripheral oxytocin administration attenuated many of these SD-induced alterations. However, because oxytocin was administered peripherally and central oxytocin levels or receptor engagement were not directly assessed, the findings cannot be interpreted as definitive evidence of direct central neuroprotection.

**Conclusion:**

The results suggest that oxytocin modulates stress responses, oxidative balance, and inflammatory pathways in aged rats subjected to chronic sleep deprivation. Although the precise central mechanisms remain unresolved, these findings support a potential role for oxytocin in mitigating SD-associated pathophysiological changes in aging and warrant further mechanistic studies to clarify its neuroprotective potential.

## Introduction

Sleep is essential for overall health and wellbeing, as it serves to restore both physical and mental functions (Alzoubi et al., 2017). Lack of adequate sleep can lead to negative outcomes such as reduced memory performance and diminished cognitive abilities (Hall et al., 2017), in addition to depression and schizophrenic symptoms that were reported by various studies. These deficits may be in part due to oxidative stress injury in the brain (Zhang et al., 2022; Choi et al., 2024). The abnormal generation of ROS may increase the risk of various neurodegenerative diseases (Maurya et al., 2021). Sleep ameliorates reactive oxygen species (ROS) generation during the sleep/wake cycle. Therefore, sleep deprivation may exacerbate oxidative stress and damage in the frontal cortex and hippocampal areas (Bin Heyat et al., 2022), confounding synaptic plasticity and long-term potentiation (LTP), and leading to memory impairments and other symptoms (Havekes and Abel, 2017).

Aging is a complex and multifactorial process involving progressive changes at the molecular, cellular, and genetic levels (Li Y. et al., 2024). The resulting decline in physiological integrity and overall functional capacity increases susceptibility to metabolic disorders, cardiovascular conditions, neurodegenerative diseases, and various forms of cancer (Zhang et al., 2025). Additionally, sleep disturbances may contribute to increased cellular aging in later life stages (Hafycz and Naidoo, 2019). Sleep problems are not age-related; however, their occurrence may increase. Sleep difficulties in elderly adults are linked to memory loss, chronic medical conditions, psychological disorders, decreased physical activity, reduced melatonin secretion, poor sleep habits, and poorer overall health (Madan Jha, 2023).

Oxytocin, a hormone produced in the hypothalamus (Moss et al., 2025) and released into the circulation via the posterior pituitary, is known to play crucial roles in uterine contractions, milk ejection, maternal behavior, and neuromodulation (Uvnäs-Moberg, 2024). It exerts neuroprotective effects in stroke by modulating γ-aminobutyric acid (GABA) signaling (Kaneko et al., 2016) and demonstrates anti-inflammatory activity by suppressing lipopolysaccharide (LPS)-induced inflammation in microglial cells (Yuan et al., 2016). It protects against cardiac and ovarian ischemia/reperfusion injury through its antioxidant properties (Houshmand et al., 2009; Akdemir et al., 2014). Moreover, oxytocin attenuates atherosclerosis (Buemann and Uvnäs-Moberg, 2020; Ko et al., 2025). It has a significant impact on social cognition, communication, interaction, appetite, pain perception, and interpersonal trust (Yao and Kendrick, 2025). Interestingly, oxytocin has been shown to alleviate oxidative stress and inflammation induced by LPS in testicular tissue, while also restoring altered sperm count, motility, and morphology. The anti-inflammatory, antioxidant, and antiapoptotic actions of oxytocin may be responsible for the therapeutic promise displayed by this compound (El-Sherbiny et al., 2024).

Considering the mounting evidence connecting sleep deprivation to oxidative stress, neuroinflammation, amyloidogenic signaling, and cognitive decline in the elderly, we assert that chronic sleep deprivation triggers a stress-induced neuroinflammatory and apoptotic cascade in the aging brain, and that oxytocin mitigates these effects. This hypothesis was tested by involving presenilin-1 (Psen1) and the serotonin 2A receptor (Htr2a) as key molecular targets. Psen1 was chosen due of its association with amyloidogenic signaling (Bagaria et al., 2022). Because of its function in the serotonergic regulation of sleep, stress, memory, and cognition, Htr2a was chosen (Guiard and Di Giovanni, 2015; Zhang and Stackman, 2015). To give a comprehensive evaluation of oxytocin’s neuroprotective mechanism, these targets were investigated in conjunction with indicators of oxidative stress, neuroinflammation, apoptosis, and oxytocin receptor signaling.

## Materials and methods

### Induction of chronic sleep deprivation

**FIGURE 1:**
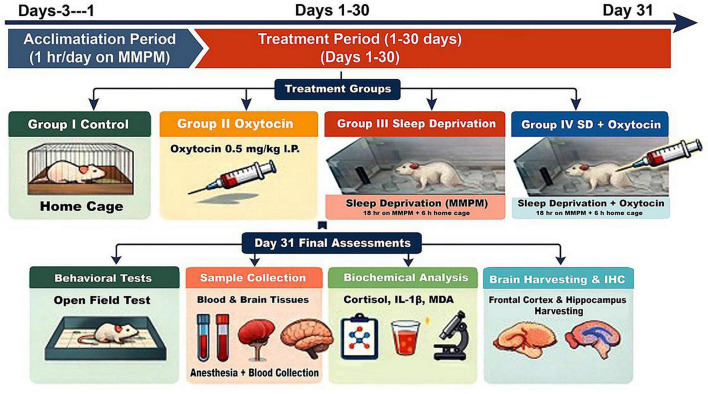
Schematic representation of the experimental timeline and study design.

The overall experimental design and timeline are summarized schematically in Figure 1. As described elsewhere, sleep deprivation (SD) in rats was induced using the Modified Multiple Platform Method (MMPM) (Suresh et al., 2021). The device, called the Modified Multiple Platform Instrument (MMPI), was made up of 12 platforms, each measuring 110 × 44 × 45 cm and having a diameter of 5.5 cm and a height of 6.0 cm. Water encircled the platforms and descended to a depth of around 1 cm. When a rat attempted to sleep, it fell into the water due to a loss of muscle tone before climbing back onto the platform. Prior to SD, the rats spent 3 days in a row acclimating to the MMPI for 1 h each day. For the SD protocol, rats were kept for 30 days, spending 6 h (08:00–14:00) in their home cages and 18 h (14:00–08:00) on the MMPI each. The animals were housed on a 12:12 h light-dark cycle and had free access to food and drink.

Rats were acclimatized to the modified multiple platform instrument (MMPI) for 3 days before experimentation. Animals were then subjected to a 30-day experimental period during which chronic sleep deprivation was induced using the modified multiple platform method (18 h/day on MMPI, 6 h/day in home cages). Oxytocin-treated groups received daily intraperitoneal injections of oxytocin (0.5 mg/kg) throughout the experimental period. On day 31, behavioral assessment was performed using the open field test, followed by anesthesia, blood collection, and harvesting of frontal cortex and hippocampal tissues for biochemical, molecular, histopathological, immunohistochemical, and multivariable analyses.

### Experimental procedure

Male Sprague Dawley rats aged 20–24 months were divided into four groups at random (*n* = 10 per group). Group I served as the normal control, whereas Group II was given daily intraperitoneal injections of oxytocin at a dose of 0.5 mg/kg for 30 days. The MMPM approach was used to induce chronic sleep deprivation in Group III (SD) for 30 days. Group IV was subjected to sleep deprivation using the MMPM method along with daily oxytocin. The intraperitoneal route was used to ensure continuous systemic exposure over long periods of time while minimizing the stress related to repeated intracerebral or intranasal administration. In low to moderate doses, peripheral injection of oxytocin may improve its translocation across the blood-brain barrier (Ermisch et al., 1985; Higashida et al., 2022). Even modest quantities of oxytocin can bind to oxytocin receptors in the brain, initiating a feed-forward loop that increases endogenous oxytocin synthesis (Fan et al., 2023).

### Ethical considerations

All the research methods adhered to globally accepted guidelines for laboratory animal care and use, and ethical permission was granted by AlMaarefa University, Riyadh, Saudi Arabia (IRB No. 23-027) and Princess Nourah bint Abdulrahman University, Riyadh, Saudi Arabia (IRB No. 23-0066). All procedures were performed by qualified personnel with special emphasis to reducing stress and discomfort in accordance with the Replacement, Reduction, and Refinement (3Rs) principles, and their health and wellbeing were observed daily. After the behavioral assessments were completed, the animals were euthanized to obtain tissue samples. Anesthesia was induced by intraperitoneal injection of a ketamine-xylazine cocktail (ketamine: 90 mg/kg; xylazine: 10 mg/kg). After confirming a surgical plane of anesthesia with the absence of pedal and corneal responses, euthanasia was carried out by decapitation.

### Assessment of animals’ behavior

Anxiety-like behavior, exploratory inclinations, and locomotor activity were assessed using the Open Field Test (OFT) (Rajizadeh et al., 2020). The OFT setup was a 120 × 120 × 40 cm wooden box. After being positioned in the middle of the arena, each rat was given 5 min to freely explore. The number of squares crossed at this time was used to measure locomotor activity. Anxiety-like behavior and exploratory behavior were observed during the test with regard to grooming and the quantity of entries into the center, respectively. Following each test, an alcohol solution was used to wipe the floor of the apparatus, which was then left to dry.

### Biochemical analysis

Following the behavioral assessments, the rats were anesthetized, and blood samples were collected. The samples were then centrifuged to obtain serum for further analysis. Serum cortisol (SL1198Ra, assay range 3–120 ng/mL, Intra-Assay: CV < 12%, Inter-Assay: CV < 12%) and interleukin-1β (IL-1β, SL0392Ra, assay range 3 pg/mL-200 pg/mL, Intra-Assay: CV, Inter-Assay: CV < 12%, 10 fold dilution) concentrations were measured according to the manufacturer’s instructions (SunLong Biotech, China). Additionally, the manufacturer’s instructions for spectrophotometric measurement of the oxidative stress marker malondialdehyde (MDA) (MD2529, Biodiagnostics, Egypt) were followed. Each sample was analyzed twice.

### Brain tissue collection

Tissue samples were obtained from the frontal cortex and the hippocampus. One portion was quickly frozen in liquid nitrogen and preserved at -80°C for later gene and protein analysis, while the other was fixed in buffered formalin for histological and immunohistochemical evaluation.

### Gene expression analysis using real-time PCR

The RNeasy Mini Kit (74104, Qiagen, Hilden, Germany) was used to extract total RNA from brain tissue samples. Absorbance at 260 nm was used to measure RNA concentration, and the 260/280 and 260/230 ratios were used to assess purity. Using the RevertAid First Strand cDNA Synthesis Kit (K1622, Thermo Scientific) and the manufacturer’s instructions, 2 μg of RNA was reverse transcribed into cDNA. To measure the expression of Htr2a and Psen1, the synthesized cDNA was amplified using SYBR Green qPCR Master Mix (Qiagen) on a real-time PCR system (Bio-Rad CFX OPUS 96). The reaction mixture (20 μL) included 2 μL of cDNA, 2 μL of forward and reverse primers, 10 μL SYBR Green Master Mix, and 6 μL nuclease-free water. The housekeeping gene was glyceraldehyde-3-phosphate dehydrogenase (GAPDH). Table 1 contains primer sequences. Relative quantification (RQ) was determined using the Double Delta Ct Method, which is expressed as RQ = 2−ΔΔCt.

**TABLE 1:** Sequences for primers used.

Primer	Sequence		Accession number
Htr2a	Forward	5′-CAT ATC TGT AGG TAT ATC CAT GCC A-3′	NM_017254.1
	Reverse	5′-AAA GTT GTC ATC GGC AAG C-3′	
Psen1	Forward	5′-GCA CCT TTG TCC TAC TTC C-3′	NM_019163.4
	Reverse	5′-TTG ATT GTC ATT CTG GCT ACG-3′	
GAPDH	Forward	5′-GTC GGT GTG AAC GGA TTT GG-3′	NM_017008.4
	Reverse	5′-TCC CGT TGA TGA CCA GCT TC-3′	

The PCR procedure was run with 44 cycles of 10 s at 95°C, 30 s at 58°C, and 20 s at 72°C. Dissociation reaction graphs were analyzed to confirm the PCR product’s specificity. A single peak indicated that one DNA sequence was amplified during PCR. The 2–11Ct method was used to measure mRNA expression levels. The results were ascertained by comparing the levels of target mRNAs to the housekeeping gene GAPDH.

To ensure expression stability, GAPDH Ct values were compared across all experimental groups prior to ΔΔCt normalization. The observed variability was negligible, and statistical analysis showed no significant variations in GAPDH Ct values across groups.

### Western blot analyses

Frontal cortex and hippocampal tissues were homogenized in RIPA buffer supplemented with protease and phosphatase inhibitor cocktails. Protein concentration in the lysates was determined using the BCA assay. Equal amounts of protein were subjected to electrophoretic separation and transferred onto a cellulose membrane. The membrane was blocked and then incubated with a primary antibody against Oxytocin Receptor (Abcam, ab300443). After washing, membranes were incubated with the corresponding secondary antibodies. The immunoreactive bands were visualized using enhanced chemiluminescence (ECL), and band intensities were quantified by densitometry. Western blots were performed in three independent technical replicates, and densitometry analysis was normalized to β-actin to account for loading variability.

### Enzyme-linked immunosorbent assay

Levels of NLRP3 (antibodies, United States, A303739), Caspase-1 (E-EL-R0371), IL-1β (E-EL-R0012), and TNF-α (E-EL-R2856) were assessed in brain tissue homogenates employing commercially available ELISA kits following manufacturers’ instructions (Elabscience, United States).

### Histopathological and immunohistochemical assessment

After processing and embedding fixed brain tissues in paraffin, 4–6 μm slices were made from the embedded blocks. Following deparaffinization and rehydration using a graded alcohol series, the sections were stained with haematoxylin and eosin (H&E) to evaluate morphological alterations under an Olympus BX-63 light microscope. ImageJ^®^ software (Wayne Rasband, NIH, Bethesda, MA, United States) was used to measure the mean number of necrotic and pyknotic cells at a magnification of × 400. Each animal had two sections, for a total of 16 sections per group. Two non-overlapping fields were evaluated for each section, for a total of 32 fields per group.

For immunohistochemical analysis, brain tissue sections were deparaffinized and rehydrated. Non-specific binding sites were blocked, and the sections were then incubated overnight with primary antibodies against Bcl-2 (Servicebio, GB114830), Caspase-3 (ABclonal, A11953), IL-1β (Servicebio, GB12113), NF-κB (Bioss, Bs-20159R), and GFAP (rabbit polyclonal, ab7260, Abcam, Cambridge, United Kingdom). Following incubation, biotinylated secondary antibodies were applied to the sections after they had been cleaned. Avidin-biotin peroxidase was used to visualize the immune response, and diaminobenzidine was used as the chromogen (Vectastain ABC-HRP kit, Vector Laboratories). Two seasoned pathologists who were unfamiliar with the experimental procedures assessed the mean area percentage of positive immunoreactivity. ImageJ^®^ software (Wayne Rasband, NIH, Bethesda, MA, United States) was used to calculate the mean area% at a magnification of × 100. Three non-overlapping fields were evaluated for each of the two parts per animal (a total of 16 sections per group), for a total of 48 fields per group.

IHC images were quantitatively assessed using the Allred score. The Allred index is assigned on a scale of 0–8, with 0–1 denoting negative, 2–3 denoting mild, 4–6 denoting moderate, and 7–8 denoting very positive. The staining intensity grading (0–3) and the percentage of positive cells grading (0–5) are added to determine the score (Fedchenko and Reifenrath, 2014). The results are quantified using the QuPath application (0.1.2) (Bankhead et al., 2017). IHC quantification was carried out using two sections per animal (16 sections/group) at a magnification of × 400. From each part, three unique, non-overlapping fields were examined (48 fields per group).

### Multivariable analyses

Multivariable analyses, such as principal component analysis (PCA), clustering heatmaps, variable importance in projection (VIP) scores, and volcano plots, were conducted using RStudio (2023.03.1 + 446 “Cherry Blossom” Release; build 6e31ffc3ef2a1f81d377eeccab71ddc11cfbd29e, 2023–05-09) on Windows with R version 4.0.2.

### Statistical analysis

The data is presented as mean ± standard error of the mean to show the precision of group mean estimates. *P* < 0.05 was regarded as significant, and all statistical tests were two-tailed. One-way Analysis of Variance (ANOVA) and Tukey’s *post-hoc* test were used for group comparisons.

## Results and discussion

### Effect on animals’ behavior

**FIGURE 2:**
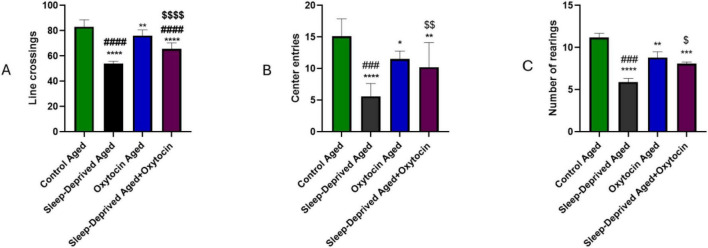
Graphical data from the Open Field Test demonstrating the effect of oxytocin on **(A)** locomotor activity, **(B)** exploratory behavior, and **(C)** anxiety-like behavior in various experimental groups. *p < 0.05, **p < 0.01 ***p < 0.001, ****p < 0.0001 vs. control aged group, ###*p* < 0.001, ####*p* < 0.0001 vs. oxytocin aged group, and ^$^*p* < 0.05, ^$$^*p* < 0.01, ^$$$$^*p* < 0.0001 vs. sleep-deprived aged group.

According to data from the Open Field Test, sleep deprivation had a negative impact on locomotor and exploratory behaviors, as evidenced by a significant decrease in the number of rearings and lines crossed, as well as an increase in anxiety-like behavior, as evidenced by a decrease in the amount of time spent in the arena’s center. On the other hand, administering oxytocin to sleep-deprived mice improved behavioral outcomes (Figure 2). Interestingly, even in the absence of sleep deprivation, oxytocin administration significantly increased locomotor and exploratory activity in older rats. These findings align with oxytocin’s anxiolytic and stress-buffering properties (Zhang et al., 2023).

The animals subjected to this platform experienced heightened anxiety, depressive behaviors, and cognitive impairment (Diao et al., 2023; Li Y. et al., 2024). On the other hand, increasing oxytocin levels demonstrated anti-stress activity in a rat model with acute stress (Tsukada et al., 2021). Oxytocin modulates central nervous system functions. Beyond its functions in regulating social and maternal behaviors, oxytocin also exhibits anxiolytic and anti-stress properties (Carter et al., 2020). The present study focused on aged rats to model the heightened susceptibility of the aging brain to chronic sleep deprivation and its associated neuroinflammatory and cognitive impairments. However, this design does not allow direct age-related comparisons. Notably, parallel experiments employing identical protocols in young and middle-aged rats are currently underway to determine whether oxytocin’s neuroprotective effects are age-dependent or conserved across the lifespan.

### Effect on systemic inflammation, oxidative stress, and serum cortisol level

**FIGURE 3:**
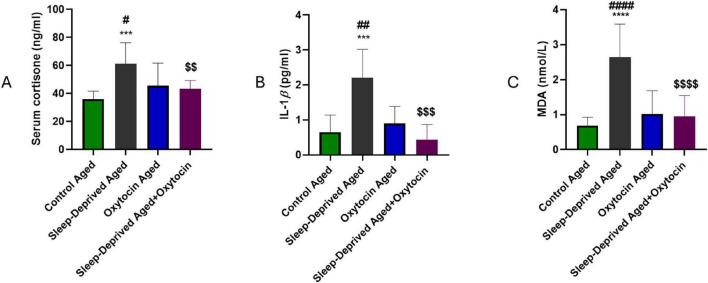
serum levels of **(A)** cortisol, **(B)** IL-1β, and **(C)** MDA across all experimental groups. ***p < 0.001, ****p < 0.0001 vs. control aged group, #*p* < 0.05, ##*p* < 0.01, ####*p* < 0.0001 vs. oxytocin aged group, and ^$$^*p* < 0.01, ^$$$^*p* < 0.001, ^$$$$^*p* < 0.0001 vs. sleep-deprived aged group.

Serum biochemical analysis revealed that cortisol levels, a glucocorticoid hormone released in response to stress, were significantly higher in the SD aged group compared to the age-matched control group (Figure 3A). Additionally, a significant increase in the inflammatory cytokine IL-1β, a proinflammatory cytokine that plays a key role in neuroinflammation, was observed (Figure 3B). In parallel, levels of MDA, a well-established biomarker of lipid peroxidation, were markedly higher in the SD aged group compared to the control aged group (Figure 3C). This increase reflects an enhancement in oxidative stress, leading to the subsequent degradation of membrane lipids. Oxytocin administration to the SD group resulted in a marked attenuation of all three markers—IL-1β, MDA, and cortisol—compared to the untreated sleep-deprived aged group. Cortisol serves as a marker of the hypothalamic-pituitary-adrenal (HPA) axis, and higher levels suggest increased physiological stress and possible disruption of circadian rhythm regulation, both of which are observed with chronic sleep deprivation (Knezevic et al., 2023). Blocking the brain oxytocin receptor activates the HPA axis and the release of corticosteroids into the blood (Neumann et al., 2000). Treatment with an oxytocin receptor antagonist resulted in a significant elevation in HPA axis activity during stress exposure relative to animals given saline. This indicates that endogenous oxytocin helps regulate and limit cortisol secretion during stress (Cavanaugh et al., 2016). Furthermore, Paksoy et al. reported that oxytocin administration decreased markers of oxidative stress and inflammation in blood samples from rats with experimental periodontitis (Paksoy et al., 2022). In a doxorubicin-induced cardiomyopathy model, oxytocin was shown to reduce systemic oxidative stress and inflammation more effectively than liraglutide and granulocyte colony-stimulating factor (Taşkıran et al., 2019).

### Effect on cellular structure

**FIGURE 4:**
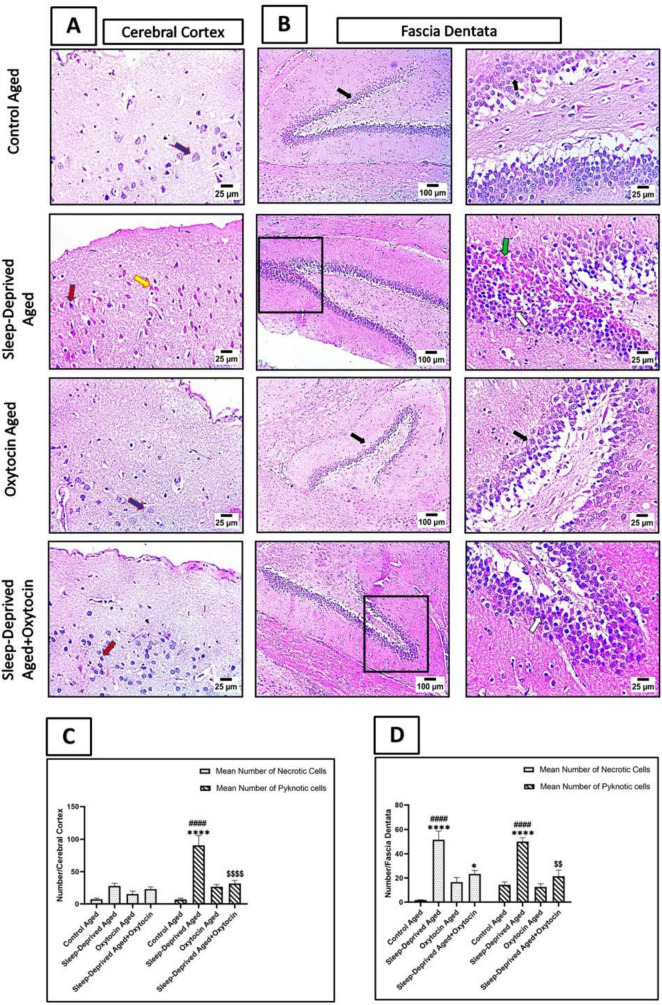
**(A)** The microscopic H&E-stained sections showing a few necrotic neurons of the frontal lobe of the cerebral cortex (blue arrow) in the control aged group and oxytocin aged group. Meanwhile, cerebral cortical sections from the sleep-deprived aged group show an increased number of necrotic neurons (yellow arrow) and a high number of shrunken and degenerated neurons with severe nuclear pyknosis (red arrow). Cerebral cortical sections from the treated sleep-deprived aged + oxytocin group show a few shrunken and degenerated neurons with pyknotic neurons (red arrow). **(B)** Microscopic H&E-stained sections showing a few necrotic neurons of the fascia dentata (black arrow) in the control aged group and oxytocin aged group. Meanwhile, cerebral cortical sections from the sleep-deprived aged group show an increased number of necrotic neurons (green arrow) and a high number of shrunken and degenerated neurons with severe nuclear pyknosis (white arrow). Cerebral cortical sections from the treated sleep-deprived aged + oxytocin group show a few shrunken and degenerated neurons with pyknotic neurons (white arrow). Magnification X: 100 bar 100 and high magnification X: 1,000 bar 25. **(C,D)** Graphs showing the mean number of necrotic and pyknotic cells in the cerebral cortex and fascia dentata. Data are expressed as mean ± SEM. *p < 0.05, ****p < 0.0001 vs. control aged; ####*p* < 0.0001 vs. oxytocin aged group and ^$$^*p* < 0.01, ^$$$$^*p* < 0.0001 vs. sleep-deprived aged group.

As shown in Figure 4, cerebral cortical and fascia dentata sections from the sleep-deprived aged group show an increased number of necrotic neurons and a high number of shrunken and degenerated neurons with severe nuclear pyknosis. However, sections from the oxytocin-treated sleep-deprived aged group show a few shrunken and degenerated neurons with pyknotic nuclei.

### Effect on oxytocin receptor

**FIGURE 5:**
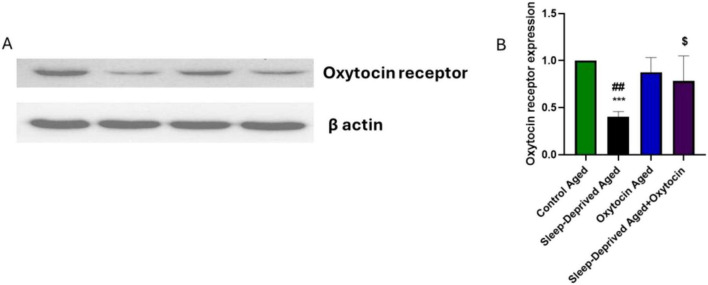
Representative Western blot images and densitometric quantification of oxytocin receptor (OXTR) protein levels, normalized to β-actin. ***p < 0.001 vs. control aged group, ##*p* < 0.01 vs. oxytocin aged group, and ^$^*p* < 0.05 vs. sleep-deprived aged group.

Figure 5 demonstrates a marked reduction in oxytocin receptor expression in brain tissue homogenates of the sleep-deprived aged group compared to age-matched controls. Administration of oxytocin to sleep-deprived rats partially restored receptor levels, suggesting that oxytocin may enhance receptor expression under stress conditions. These results align with previous research linking stress and disrupted sleep to impaired oxytocin signaling. For example, Li et al reported reduced oxytocin receptor mRNA in a chronic social defeat model. Oxytocin administration can reverse this effect, influencing presynaptic plasticity and sleep response (Li et al., 2021). Decreased oxytocin receptor expression has also been reported in chronically stressed mice exposed to maternal separation, social defeat, and social isolation (Jeon et al., 2024). Moreover, hypermethylation of the oxytocin receptor gene has been linked to different stress models (Bey et al., 2022) and shows an inverse relationship with cortisol levels (Wiley et al., 2023). Although peripheral oxytocin administration in this investigation was associated with increased OXTR protein levels in the brains of sleep-deprived rats, these findings require careful interpretation. Because intraperitoneal oxytocin cannot easily penetrate the blood-brain barrier, the observed changes could be attributed to indirect effects on the periphery or stress-induced changes, rather than direct interaction with central receptors. Previous research has shown that persistent oxytocin therapy during stress or disruptions in homeostasis can impact OXTR expression. Human investigations show that frequent intranasal oxytocin treatment leads to decreased OXTR DNA methylation, an epigenetic marker linked with increased receptor availability (Moerkerke et al., 2024). Many factors influence OXTR expression dynamics, including developmental stage, sex, species, and experience events, emphasizing the multidimensional nature of receptor modulation following persistent oxytocin exposure (Vaidyanathan and Hammock, 2017). As a result, our findings suggest that oxytocin may have an effect on receptor modification, however this is not conclusive. More research into central oxytocin bioavailability and receptor binding is required for complete mechanistic validation.

### Effect on Psen1 and Htr2a

**FIGURE 6:**
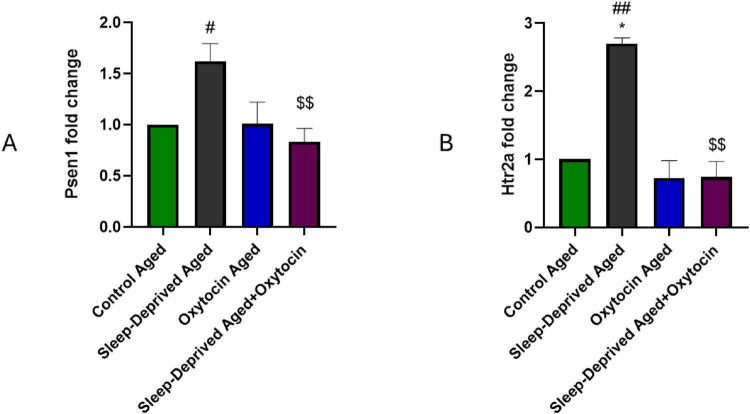
Bar graphs of qPCR analysis shows mRNA expression levels of **(A)** Psen1 and **(B)** Htr2a in brain tissue. *p < 0.05, vs. control aged group, #*p* < 0.05, ##*p* < 0.05 vs. oxytocin aged group, and ^$$^*p* < 0.01 vs. sleep-deprived aged group.

The data in Figure 6 illustrates the effect of sleep deprivation on gene expression of Psen1 and Htr2a. Sleep deprivation in aged animals led to a marked increase in Psen1 and Htr2a gene expression. However, oxytocin administration to sleep-deprived aged animals results in the restoration of gene expression to levels comparable to the control aged group. Psen1 is a key component of the γ-secretase complex, an intermediate protease responsible for cleaving several type I transmembrane proteins, most notably the amyloid precursor protein (Bagaria et al., 2022). Mutation or dysregulation of Psen1 is strongly linked to neurodegeneration due to its role in producing the amyloid β protein (Orticello et al., 2023). Ju et al., reported increased cerebrospinal fluid amyloid-β levels in sleep-deprived participants, suggesting that sleep deprivation may promote amyloid plaque buildup and downstream pathologies leading to neurodegeneration and Alzheimer’s disease, such as tauopathy and inflammation (Ju et al., 2017). Furthermore, sleep restoration has been found to enhance amyloid β clearance and improve neuropathological and behavioral deficits in an Alzheimer’s disease model (Zhao et al., 2023). Interestingly, oxytocin intervention in socially isolated transgenic Alzheimer’s disease models, which coexpress amyloid precursor protein and Psen1, significantly reduced amyloid β plaque accumulation, decreased microglial activation, and mitigated behavioral deficits (Li et al., 2025a). Although Psen1 is directly involved in amyloidogenic processing, the present study did not assess amyloid-β levels or plaque formation, and thus the functional implications of Psen1 modulation on amyloid pathology remain to be determined. Htr2a plays a crucial role in regulating mood, cognition, and sleep (Elmenhorst et al., 2012). Previous studies demonstrated increased Htr2a mRNA levels with sleep deprivation (Maple et al., 2015; Zhao et al., 2022). Notably, evidence of functional crosstalk exists between serotonin and oxytocin systems. This interaction suggests that oxytocin may buffer the effects of stress-induced Htr2a upregulation (Mottolese et al., 2014). The increase in Htr2a expression generated by sleep deprivation may be a serotonergic system homeostatic response to maintain brain homeostasis under prolonged stress. Oxytocin therapy lowered Htr2a levels, indicating a reestablishment of serotonergic homeostasis and possibly contributing to the observed behavioral improvements. Nonetheless, the specific functional ramifications of Htr2a regulation are complex and warrant more investigation.

### Effect on tissue inflammation

**FIGURE 7:**
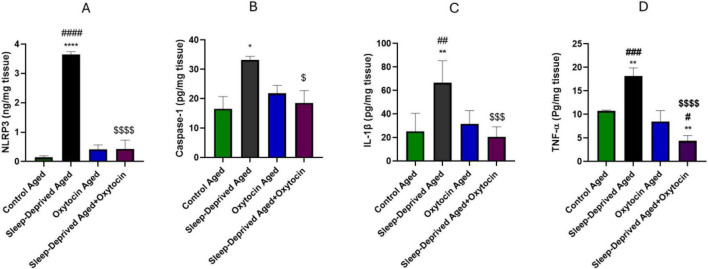
Bar graphs representing concentrations of neuroinflammatory markers, **(A)** NLRP3, **(B)** Caspase-1, **(C)** IL-1β, **(D)** TNF-α in brain tissue homogenates as measured by ELISA. *p < 0.05, **p < 0.01 ****p < 0.0001 vs. control aged group, #*p* < 0.05, ##*p* < 0.01, ###*p* < 0.001, ####*p* < 0.0001 vs. oxytocin aged group, and ^$^*p* < 0.05, ^$$$^*p* < 0.001, ^$$$$^*p* < 0.0001 vs. sleep-deprived aged group.

Figure 7 showed a significant increase in NLRP3, Caspase-1, IL-1β, and TNF-α levels in brain tissue homogenates from the sleep-deprived aged group compared to the control aged group. Oxytocin treatment led to a notable reduction in the levels of these inflammatory markers in brain tissue relative to the sleep-deprived aged group.

**FIGURE 8:**
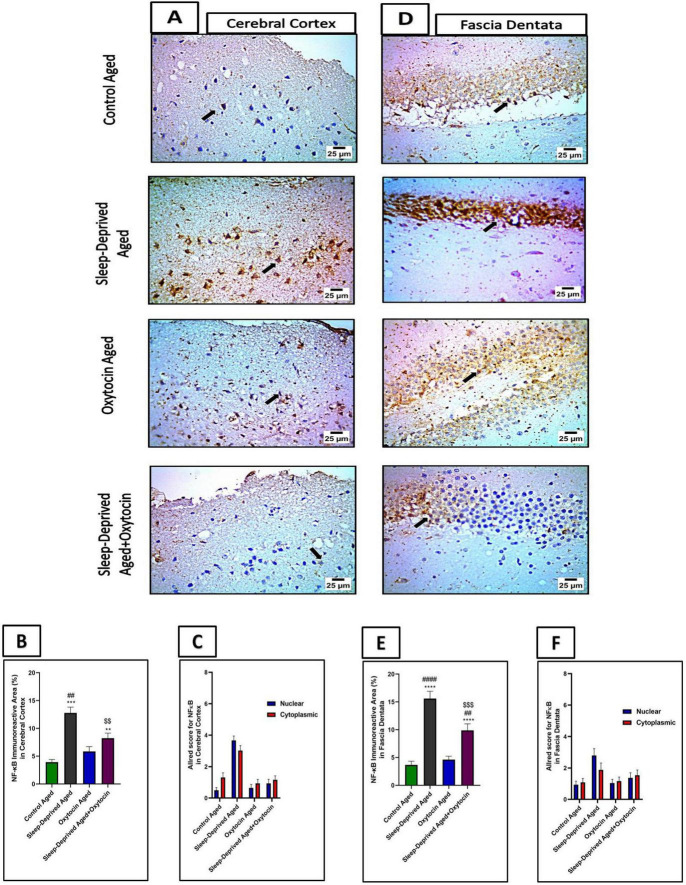
**(A)** The microscopic immunostained sections of the frontal lobe of the cerebrum from the control aged group and oxytocin aged group show mild expression in neurons (black arrow) against NF-κB. Meanwhile, cerebral cortical sections from the sleep-deprived aged group show strong positive expression (black arrow) against NF-κB. Cerebral cortical sections from the sleep-deprived aged + oxytocin group show decreased positive expression in neurons (black arrow) against NF-κB. High magnification IHC counterstained with Mayer’s hematoxylin. X: 1,000 bar 25. **(B)** The graph shows immunoreactive area (%) for NF-κB expression in the cerebral cortex. Data are expressed as mean ± SEM. ***p < 0.001 and **p < 0.01 vs. control aged group, ##*p* < 0.01 vs. oxytocin aged group, and ^$$^*p* < 0.01 vs. sleep-deprived aged group. **(C)** The graph shows the Allred score for cytoplasmic and nuclear NF-κB expression in the cerebral cortex. The graph shows significant translocation of NF-κB from the cytoplasm to the nucleus in the sleep-deprived aged group. Oxytocin treatment reversed this translocation in the sleep-deprived aged + oxytocin group. Values are expressed as mean ± S.E.M. **(D)** The microscopic immunostained fascia dentata sections from the control aged group and oxytocin aged group show mild expression in neurons (black arrow) against NF-κB. Meanwhile, sections of fascia dentata from the sleep-deprived aged group show strong positive brown staining in neurons (black arrow) against NF-κB. Sections of fascia dentata from sleep-deprived aged + oxytocin show decreased positive brown staining in neurons (black arrow) against NF-κB. High magnification IHC counterstained with Mayer’s hematoxylin. X: 1,000 bar 25. **(E)** The graph shows immunoreactive area (%) for NF-κB expression in fascia dentata. Data are expressed as mean ± SEM. ****p < 0.0001 vs. control aged group, ##*p* < 0.01, ####*p* < 0.0001 vs. oxytocin aged group, and ^$$$^*p* < 0.001 vs. sleep-deprived aged group. **(F)** The graph shows the Allred score for cytoplasmic and nuclear NF-κB expression in fascia dentata. The graph shows the translocation of NF-κB from the cytoplasm to the nucleus in the sleep-deprived aged group. Oxytocin treatment improved this translocation in the sleep-deprived aged + oxytocin group. Values are expressed as mean ± S.E.M. Allred index (0–1 = negative, 2–3 = mild, 4–6 = moderate, and 7–8 = strongly positive).

**FIGURE 9:**
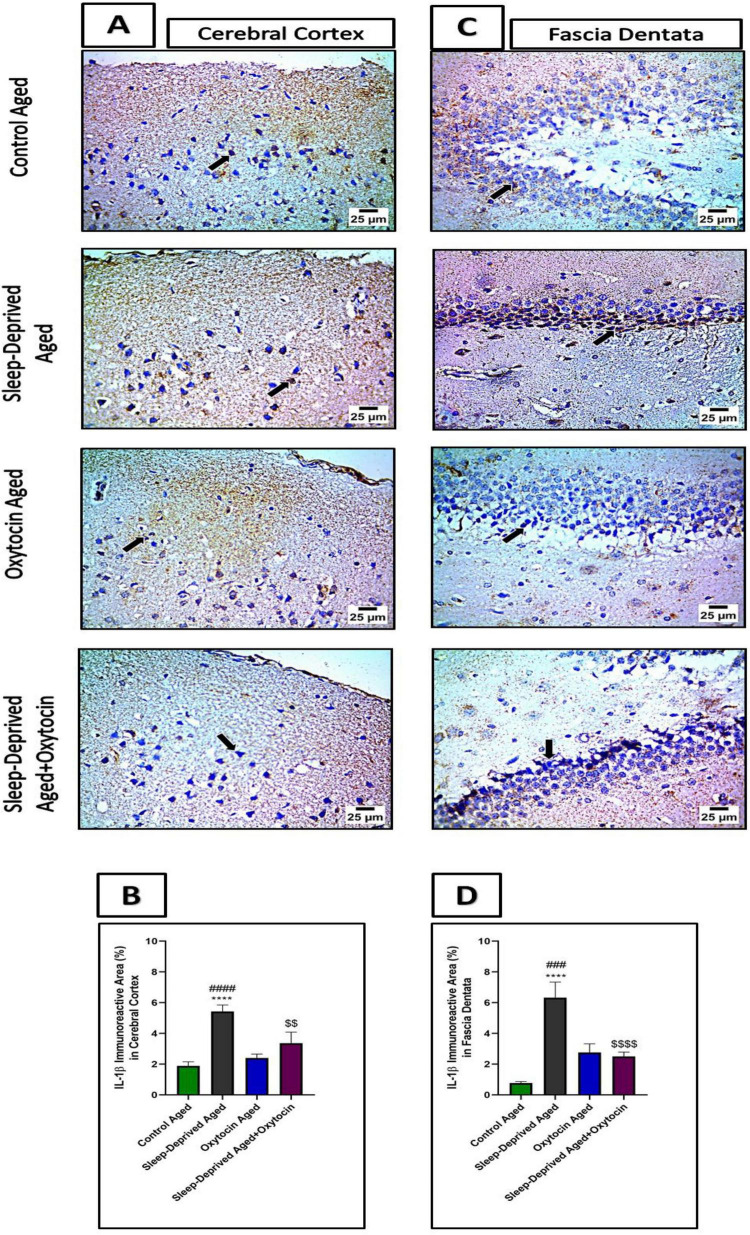
**(A)** The microscopic immunostained sections of the frontal lobe of the cerebrum from the control aged group and oxytocin aged group show moderate expression in neurons (black arrow) against IL-1β. Meanwhile, cerebral cortical sections from the sleep-deprived aged group show strong positive expression (black arrow) against IL-1β. Cerebral cortical sections from the treated sleep-deprived aged + oxytocin group show decreased positive expression in neurons (black arrow) against IL-1β. High magnification IHC counterstained with Mayer’s hematoxylin. X: 1,000 bar 25. **(B)** The graph shows immunoreactive area (%) for IL-1β expression in the cerebral cortex. Data are expressed as mean ± SEM. ****p < 0.0001 vs. control aged group, ###*p* < 0.001, ####*p* < 0.0001 vs. oxytocin aged group, and *^$$^p* < 0.01 vs. sleep-deprived aged group. **(C)** The microscopic immunostained fascia dentata sections show mild expression in neurons (black arrow) against IL-1β from the control aged group and oxytocin aged group. Meanwhile, sections of fascia dentata from the sleep-deprived aged group show strong positive brown staining in neurons (black arrow) against IL-1β. Sections of fascia dentata from sleep-deprived aged + oxytocin show decreased positive brown staining in neurons (black arrow) against IL-1β. High magnification IHC counterstained with Mayer’s hematoxylin. X: 1,000 bar 25. **(D)** The graph shows immunoreactive area (%) for IL-1β expression in fascia dentata. Data are expressed as mean ± SEM. ****p < 0.0001 vs. control aged group, ####*p* < 0.0001 vs. oxytocin aged group, and *^$$$$^p* < 0.0001 vs. sleep-deprived aged group.

These results were further validated by analysis of NF-κB and IL-1β immunostained sections, Figures 8, 9, where a significant positive expression of these markers was more evident in the frontal cortex and fascia dentata of the sleep-deprived aged group compared to the control aged group. These immunoreactivities were mitigated by oxytocin treatment in the frontal cortex and fascia dentata compared to the sleep-deprived group.

Sleep deprivation is strongly associated with neuroinflammation, and the NLRP3/Caspase-1/IL-1β pathway plays a key role in this process (Niznikiewicz et al., 2017). Chronic sleep deprivation causes oxidative stress (Davinelli et al., 2024), damages macromolecules (Carroll et al., 2016), and leads to mitochondrial dysfunction (Liu et al., 2025), all of which can activate NLRP3. When activated, NLRP3 recruits and activates Caspase-1, which then cleaves the inactive pro-IL-1β into its active form, IL-1β, a proinflammatory cytokine that promotes microglial activation, synaptic remodeling, and impairs neuronal plasticity. Inhibiting NLRP3 activation has been shown to have neuroprotective effects, reducing neuroinflammation and cognitive deficits in sleep-deprived mice (Zhou et al., 2022; Fu et al., 2024; Li et al., 2025b). Chronic sleep deprivation in rats leads to low-grade neuroinflammation, characterized by elevated hippocampal levels of inflammatory cytokines (TNF-α and IL-1β), activation of proinflammatory transcription factors (NF-κB), and increased expression of GFAP and IL-1β. The rise in GFAP indicates active gliosis, which correlates with memory impairment and anxiety-like behaviors (Manchanda et al., 2018). NF-κB is a transcription factor that regulates genes involved in inflammation. It becomes activated in response to stress signals and plays a key role in many diseases, including neuroinflammation (Babkina et al., 2021). Sleep deprivation activates NF-κB signaling, leading to increased proinflammatory cytokines in the brain and peripheral tissues. This activation leads to neuroinflammation, glial cell activation, and oxidative stress, substantially impairing neuronal function and negatively affecting cognition and mood. The resulting inflammation forms a vicious cycle that exacerbates brain dysfunction during prolonged sleep deprivation (Mahalakshmi et al., 2022; Chen et al., 2024).

**FIGURE 10:**
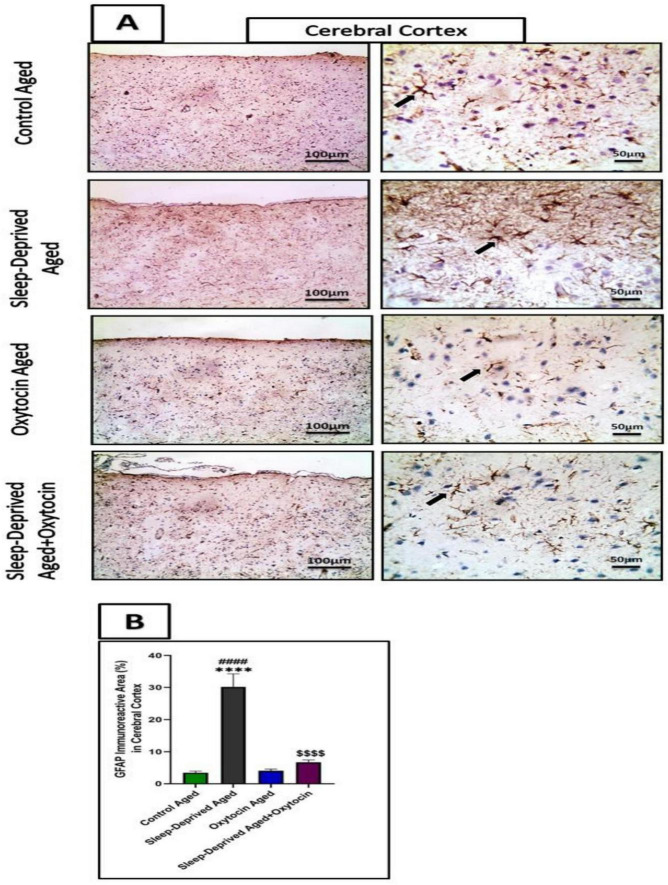
Representative microscopic immunohistochemical and quantitative images analysis show the expression and distribution of GFAP in brain tissues **(A)**: frontal lobe of the cerebrum, from all experimental groups. **(B)** The Graph shows immunoreactive area (%) for GFAP expression in the frontal lobe. Data are expressed as mean ± SEM. ****p < 0.0001 vs. control aged, ####*p* < 0.001 vs. oxytocin aged group and ^$$$$^*p* < 0.0001 vs. sleep-deprived aged group. Low magnification IHC counterstained with Mayer’s hematoxylin. X: 100 bar 100 and high magnification X: 400 bar 50.

Furthermore, GFAP immunostained sections showed significant astrogliosis, with an increased number of GFAP-positive astrocytes in the sleep-deprived aged group compared to the control aged group in the cerebral cortex. Oxytocin administration had region-specific protective effects compared to the sleep-deprived aged group in the cerebral cortex (Figure 10).

Sleep deprivation induces glial activation, and active gliosis in the brain is linked to neuroinflammation and increased levels of proinflammatory cytokines. In patients with chronic insomnia, higher serum GFAP correlates with poorer subjective sleep quality and cognitive impairment, emphasizing the role of glial activation in memory and brain function during sleep deprivation (Zhang et al., 2020). Ozathaley et al reported increased NLRP3 and GFAP in the hippocampus of sleep-deprived mice (Ozathaley et al., 2023). These findings collectively underscore the pivotal role of glial activation and NLRP-3-mediated neuroinflammation in the cognitive and neuronal impairment associated with sleep deprivation.

Under stress, oxytocin decreases the production of inflammatory cytokines by regulating the HPA axis and modulating neuroinflammatory signaling pathways (Matsushita et al., 2019; Kamrani-Sharif et al., 2023). In a rat model of neonatal hypercapnic-hypoxia injury, oxytocin reduced hippocampal gliosis and showed anti-inflammatory efficacy (Sünnetçi et al., 2021). Additionally, oxytocin dampened the expression of proinflammatory and pyroptotic factors, including NLRP3, and ameliorated neurological deficits in an experimental model of Intracerebral hemorrhage (Yang et al., 2023). Furthermore, studies in both animals and humans indicate that oxytocin plays a regulatory role in stress and related neuroinflammation, helping to alleviate stress-related behaviors (Takayanagi and Onaka, 2021).

### Effect on apoptosis

**FIGURE 11:**
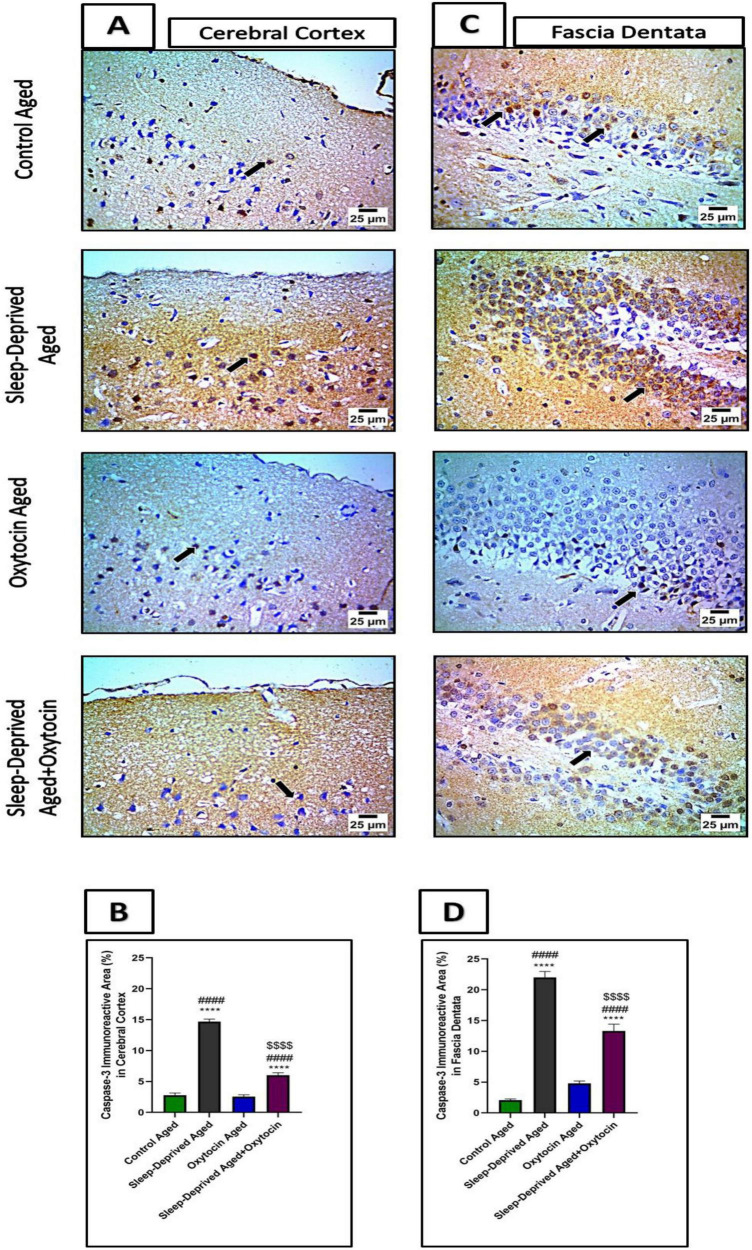
**(A)** The microscopic immunostained sections of the frontal lobe of the cerebrum from the control aged group and oxytocin aged group show moderate expression in neurons (black arrow) against caspase-3. Meanwhile, cerebral cortical sections from the sleep-deprived aged group show strong positive expression (black arrow) against caspase-3. Cerebral cortical sections of the frontal lobe from the treated sleep-deprived aged + oxytocin group show decreased positive expression in neurons (black arrow) against caspase-3. High magnification IHC counterstained with Mayer’s hematoxylin. X: 1,000 bar 25. **(B)** The Graph shows immunoreactive area (%) for caspase-3 expression in the frontal lobe. Data are expressed as mean ± SEM. ****p < 0.0001 vs. control aged group, ####*p* < 0.0001 vs. oxytocin aged group, and *^$$$$^p* < 0.0001 vs. sleep-deprived aged group. **(C)** The microscopic immunostained fascia dentata sections from the control aged group and oxytocin aged group show moderate expression in neurons (black arrow) against caspase-3. Meanwhile, sections of fascia dentata from the sleep-deprived aged group show strong positive brown staining in neurons (black arrow) against caspase-3. Sections of fascia dentata from sleep-deprived aged + oxytocin show decreased positive brown staining in neurons (black arrow) against caspase-3. High magnification IHC counterstained with Mayer’s hematoxylin. X: 1,000 bar 25. **(D)** The graph shows immunoreactive area (%) for caspase-3 expression in the fascia dentata. Data are expressed as mean ± SEM. ****p < 0.0001 vs. control aged group, ####*p* < 0.0001 vs. oxytocin aged group, and *^$$$$^p* < 0.0001 vs. sleep-deprived aged group.

**FIGURE 12:**
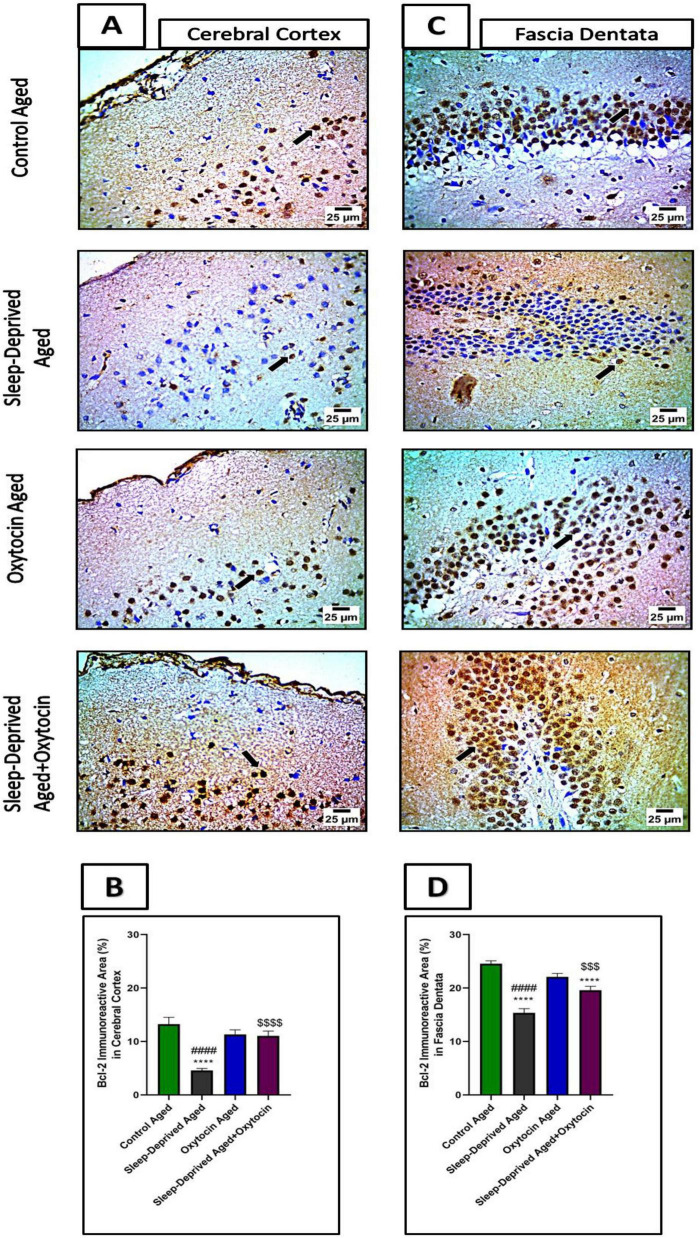
**(A)** The microscopic immunostained sections of the frontal lobe of the cerebrum from the control aged group and oxytocin aged group show moderate positive expression in neurons (black arrow) against Bcl-2. Meanwhile, cerebral cortical sections of the frontal lobe from the sleep-deprived aged group show decreased positive expression (black arrow) against Bcl-2. Frontal lobe sections from the treated sleep-deprived aged + oxytocin group show strong positive expression in neurons (black arrow) against Bcl-2. High magnification IHC counterstained with Mayer’s hematoxylin. X: 1,000 bar 25. (B) The graph shows immunoreactive area (%) for Bcl-2 expression in the cerebral cortex. Data are expressed as mean ± SEM. ^****^p < 0.0001 vs. control aged group, ####*p* < 0.0001 vs. oxytocin aged group, and ^$$$$^*p* < 0.0001 vs. sleep-deprived aged group. (C) The microscopic immunostained fascia dentata sections from the control aged group and oxytocin aged group show moderate positive brown staining in neurons (black arrow) against Bcl-2. Meanwhile, sections of fascia dentata from the sleep-deprived aged group show decreased positive brown staining in neurons (black arrow) against Bcl-2. Sections of fascia dentata from sleep-deprived aged + oxytocin show increased positive brown staining in neurons (black arrow) against Bcl-2. High magnification IHC counterstained with Mayer’s hematoxylin. X: 1000 bar 25. (D) The graph shows immunoreactive area (%) for Bcl-2 expression in fascia dentata. Data are expressed as mean ± SEM. ****p < 0.0001 vs. control aged group, ####*p* < 0.0001 vs. oxytocin aged group, and ^$$$^*p* < 0.001 vs. sleep-deprived aged group.

Immunohistochemical analysis of caspase-3 and Bcl-2 revealed an increase in caspase-3–positive areas in the frontal cortex and fascia dentata of the sleep-deprived aged group compared to controls. Treatment with oxytocin exhibited strong neuroprotective effects by reducing caspase-3 expression relative to the sleep-deprived group. Conversely, Bcl-2 immunostaining in cortical and fascia dentata neurons was significantly decreased in the sleep-deprived aged group, reflecting downregulation of this neuroprotective marker. Oxytocin administration restored Bcl-2 expression to levels comparable to controls (Figures 11, 12).

Sleep deprivation has been extensively associated with the activation of apoptosis in various brain regions, including the hippocampus and frontal cortex. This apoptotic process is primarily triggered by oxidative stress, mitochondrial dysfunction, and neuroinflammation caused by prolonged lack of sleep. Sleep deprivation has been found to induce neuronal apoptosis in the brain of zebrafish, as demonstrated by increased caspase-3 and reduced Bcl2 (Lu et al., 2023), and to increase caspase-3 expression in the hippocampus of sleep-deprived rats (Dai et al., 2021). Somarajan et al demonstrated that SD induced neuronal death in rats by activating the mitochondrial intrinsic pathway (Somarajan et al., 2016).

Asaba et al. reported that treatment with oxytocin reduced caspase-3-mediated apoptosis in a concentration-dependent manner in a model of early Alzheimer’s disease-like pathology (Asaba et al., 2025). Moreover, oxytocin hampered both mitochondrial and non-mitochondrial apoptosis, showing neuroprotection in a rat model of Huntington’s Disease (Rabie et al., 2025). These findings highlight oxytocin as a promising therapeutic agent against neurodegeneration by modulating key apoptotic pathways involved in various neurodegenerative disorders. Collectively, these results suggest that oxytocin may act upstream by reducing oxidative stress and stress-axis activation, thereby indirectly attenuating NLRP3 inflammasome activation, NF-κB signaling, and apoptosis. However, direct causality necessitates supplementary mechanistic tests. Future studies are needed to better identify the principal molecular targets of oxytocin in neurotoxicity generated by sleep loss.

**FIGURE 13:**
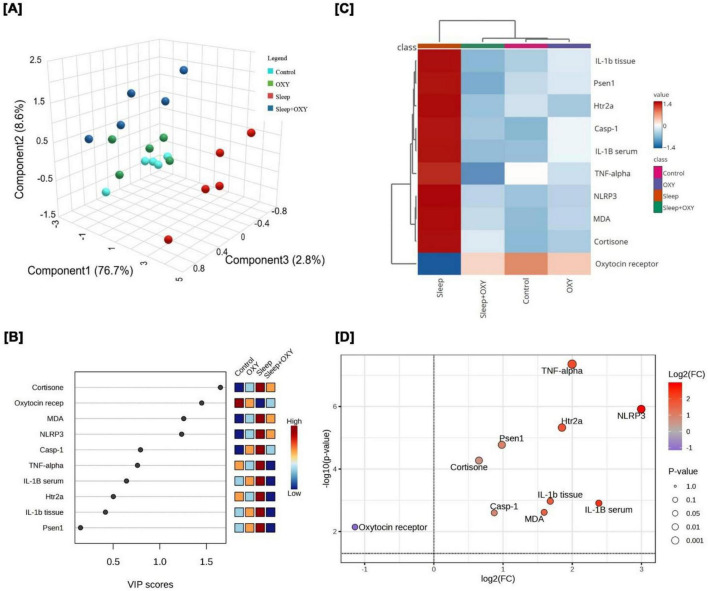
Multivariable analyses of all data sets after sleep deprivation and/or OXY intervention. **(A)** 3D plot of principal component analysis (PCA). **(B)** Variable importance score (VIP) scores declare the most influencing variable. **(C)** The correlation heatmap illustrates the inter-relationship among the studied variables. **(D)** Volcano plot among different groups. IL1B, interleukin 1B; MDA, malondialdehyde; Casp-1, Caspase-1; OXY, oxytocin; PCA, principal component analysis; Cortisone, Cortisone; Psen1, Presenilin-1; Htr2a, 5-hydroxytryptamine receptor 2A; NLRP3, Nucleotide-binding Oligomerization Domain-like Receptor Protein 3; TNF-α, tumor necrosis factor Alpha; VIP, Variable importance in projection.

To comprehensively evaluate the overall impact of sleep deprivation and oxytocin treatment on the measured biochemical and molecular parameters, a series of multivariable analyses was conducted, including PCA, VIP, hierarchical clustering, and volcano plotting. As depicted in Figure 13A, PCA was applied to determine the relationship among the different interventions. Principal component analysis (PCA) identified three principal components accounting for a total of 86.1% of the variation across all measured parameters. Component 1 contributed the largest proportion of variance (76.2%), whereas components 2 and 3 explained smaller proportions of 7.7 and 2.2%, respectively. PCA results showed a clear separation of sleep-deprived aged rats from the control aged, oxytocin-treated aged, and sleep-deprived aged + oxytocin groups. Notably, sleep-deprived aged rats that received oxytocin exhibited a distinct profile compared to those subjected to sleep deprivation alone, highlighting the modulatory effect of oxytocin. In Figure 13B, VIP scores revealed that cortisone, Oxytocin receptor, oxidative stress (MDA), Htr2a, Psen1, and inflammatory markers (NLRP3, TNF-α, IL-1β, and caspase-1) were the most influencing variables in the present study. The clustering heatmap in Figure 13C further revealed significant changes in the average concentrations of measured variables among the various experimental groups. Compared to the aged control group, oxytocin-aged and sleep-deprived aged + oxytocin groups, the aged sleep-deprived group exhibited notable injury, characterized by marked alterations in the concentration values of inflammatory markers (TNF-α, IL-1β, caspase-1, NLRP3) and oxidative stress markers (MDA). These data confirm that oxytocin may confer protection against sleep deprivation. Finally, the volcano plot in Figure 13D graphically quantifies these changes, mainly between sleep-deprived aged and oxytocin aged, control aged, and sleep-deprived aged + oxytocin, revealing a wide range of significantly altered parameters. The -log10 (*P*-value) on the Y-axis indicates the statistical significance of these changes, while the log2 (fold change) on the X-axis discriminates the upregulated (right quadrant) and downregulated (left quadrant) variables. The data points crossing the vertical and horizontal thresholds indicate that sleep deprivation exposure resulted in extensive and substantial changes. The effective counteraction of these effects occurred when sleep deprivation was co-administered with oxytocin.

## Limitations

Notable limitations should be considered when interpreting our findings. First, although elderly rats serve as a pertinent model for age-related cognitive decline, the chronic sleep deprivation model utilized in this study is more severe and persistent than typical human sleep deficit, which is generally intermittent and less severe. This constrains the direct translation of our findings to clinical populations. Second, the study exclusively involved male rats, and the lack of female animals limits generalizability due to existing sex variations in response to stress and oxytocin system function. Third, only one dose of oxytocin (0.5 mg/kg, intraperitoneally) was examined; more dose-response studies and alternate delivery routes (e.g., intranasal) are necessary to inform possible clinical translation. Fourth, the daily injections needed for long-term oxytocin use could cause stress associated with handling, which could affect behavioral and stress-related results. Lastly, the absence of a young adult control group limits the capacity to directly evaluate age-related vulnerability to sleep deprivation and oxytocin responsiveness. Nonetheless, it is envisaged that these constraints will be addressed and a comprehensive lifespan view will be provided in forthcoming studies using an identical experimental approach in young and middle-aged groups.

## Conclusion

In conclusion, chronic sleep deprivation in aged rats caused behavioral deficits, oxidative stress, systemic inflammation, neuronal damage, and activation of the apoptotic pathway. Oxytocin administration effectively counteracted these effects by restoring receptor expression, reducing inflammation, and suppressing apoptosis, hence preserving neuronal structure. These findings highlight the neuroprotective role against sleep-deprivation-induced damage. Thus, oxytocin may represent a promising therapeutic strategy for stress and age-related neurodegenerative conditions.

## Data Availability

The original contributions presented in this study are included in this article/supplementary material, further inquiries can be directed to the corresponding author.
